# Why clinical translation cannot succeed without failure

**DOI:** 10.7554/eLife.12844

**Published:** 2015-11-24

**Authors:** Alex John London, Jonathan Kimmelman

**Affiliations:** Department of Philosophy and Center for Ethics and Policy, Carnegie Mellon University, Pittsburgh, United Statesajlondon@andrew.cmu.edu; Studies of Translation, Ethics and Medicine Research Group, Biomedical Ethics Unit, McGill University, Montréal, Canadajonathan.kimmelman@mcgill.ca

**Keywords:** preclinical research, clinical trials, drug development, ethics, reproducibility, translational research

## Abstract

The high rates of attrition that occur in drug development are widely regarded as problematic, but the failure of well-designed studies benefits both researchers and healthcare systems by, for example, generating evidence about disease theories and demonstrating the limits of proven drugs. A wider recognition of these benefits will help the biomedical research enterprise to take full advantage of all the information generated during the drug development process.

For every hundred drugs introduced into clinical development, approximately 90 are never approved for clinical use, and success rates for secondary applications of licensed drugs are not much better (Hay et al., 2014). Many commentators view high rates of attrition—defined as a failure to show that a promising drug is useful—as symptomatic of inefficient drug development. Some attribute inefficiencies to flawed preclinical or clinical study design, others to regulatory impediments, and still others to deficits in scientific techniques (Kola and Landis, 2004; Collins, 2011). Attrition is therefore viewed as a burden for the research enterprise and, since development costs are reflected in the price of new pharmaceuticals, for healthcare systems too.

However, given the incompleteness of our knowledge of the relevant disease processes and pharmacology, attrition is actually a necessary part of a rational and efficient approach to building a robust understanding of the diseases we are trying to treat. Attrition is also crucial for refining how this knowledge is applied in the clinic. Indeed, recognizing that unsuccessful translation trajectories can contribute to the viability of research and healthcare systems, and understanding more about this process, should lead to improvements in both systems.

## How failure promotes translation

First, although pharmaceutical development is aimed at producing new drugs, the most valuable product of translation efforts is information about disease and drug mechanisms. This information is valuable because it informs drug development and it guides clinical practice (Kimmelman and London, 2015). For example, theories about amyloid metabolism have guided the selection of drugs and populations for the testing of Alzheimer's treatments for the last decade. About a third of all oncology prescriptions are off-label (Conti et al., 2013), and theories of tumor physiology help clinicians to extend findings from trials in order to devise treatments for patients who might not have met the initial criteria for treatment with the drug. However, our understanding of disease causation and pharmacology are incomplete, and many translational trajectories falter because the theoretical understandings guiding the trials are incorrect. (This is especially true when failures occur in phase 2 trials.) Even when disease and drug models build on robust basic science and preclinical testing, in vivo studies in humans represent the only way of confirming or discrediting emerging theories.

Second, efficient methods for generating this information limit the extent to which early signs of promise can be vindicated in the later phases of the translation process. To see why this is so, consider that drug development activities naturally divide between exploratory investigations aimed at identifying intervention candidates, and confirmatory investigations aimed at demonstrating their clinical utility (Kimmelman et al., 2014). Identifying promising interventions is akin to exploring a vast, multidimensional landscape of agents, doses, disease indications and treatment schedules. The methods used to explore this landscape (which happens during preclinical research and early-phase trials) often rely on small sample sizes and/or surrogate endpoints. This allows large areas of the landscape to be explored quickly and at relatively low cost. However, economy and speed come at a cost, since small and less rigorous studies tend to produce more false positives (i.e., studies that show spurious clinical promise due to bias or random variation) (Button et al., 2013).

**Figure 1:**
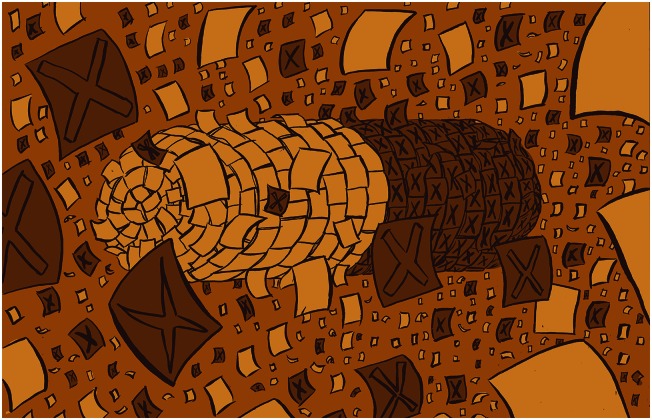
Negative results advance drug discovery by helping to improve our understanding of both diseases and treatments.

Illustration: Claudia Stocker, vividbiology.com.

Third, the rational desire to allocate resources to interventions that show the greatest promise entails a certain amount of attrition. In particular, base rates for discovering truly effective interventions are likely to be low in areas where our knowledge of disease process, mechanism and pharmacology are underdeveloped. As is well known in diagnosis, when base rates are low, false positive tests due to random variation are frequent, even if tests are sensitive. Similarly, most promising results in early-phase studies in novel areas will prove to be false positives. Drug development thus confronts the problem of ‘winners curse’ (Button et al., 2013). This refers to the tendency of randomly large effects to regress toward a mean on subsequent testing. Consequently, large treatment effects in phase 1 studies will tend to diminish on testing in phase 2, and similarly for large effects seen in phase 2 trials. Several studies have shown such statistical tendencies in clinical research (Ioannidis et al., 2001). Winner's curse is the result of an appropriate type of experimenter bias, whereby researchers follow up on treatments that show large effects and set aside treatments that show smaller effects.

Fourth, ethical constraints on drug trials mean that new interventions will frequently fail to prove superior to standard treatments. This happens because enrolling patients with serious illnesses into randomized trials is only ethical where there is genuine and evidence-based uncertainty within the expert clinical community about the comparative advantage of the interventions in a trial. This condition, which is known as clinical equipoise (London, 2007), serves two functions. First, it ensures that patients can enter trials knowing they are receiving competent care (Freedman, 1987). Second, it promotes efficiency because it ensures that randomized trials are conducted only where there is genuine uncertainty about the value of a new treatment. If the uncertainty in the expert community is based on accurate evidence, novel interventions should prove superior to standard treatments as often as standard treatments prove superior to novel interventions. Indeed, studies have shown such patterns (Djulbegovic et al., 2013), suggesting that early-phase research provides a reliable basis for designing randomized trials, and that the clinical research enterprise adequately protects the welfare of subjects.

Finally, even when drugs with high clinical promise are identified, the process of refining our understanding of how to use them requires some level of failure. Drugs are only clinically useful insofar as clinicians know which dose, schedule, timing and diagnostic eligibility criteria to apply at the bedside. For example, some drugs will be ineffective for patients below a certain diagnostic score and/or toxic for patients who show a higher value (Schmidt, 2011; Kimmelman, 2012). These limits on the clinical utility of an intervention often cannot be defined without testing regimens that prove to be outside the desired window of activity. But without such failures and negative results, clinicians lack the knowledge of how far they can extend the application of a new drug while preserving its desirable effects. Consequently, efficient healthcare—even for successfully translated drugs—depends on testing regimens that fail, but that help to define the window within which a drug can be used safely and effectively.

## Learning from failure

Obviously, some failures—such as studies that fail to recruit a meaningful sample, studies that suffer fatal design flaws, or studies that fail to deal with random variation and bias (Yusuf et al., 1984)—are unmitigated failures because they are not likely to deliver any useful information. Nevertheless, the foregoing analysis suggests that many unsuccessful translation trajectories are a product of efficient, moral and rational research efforts rather than inattention to experimental design. Well-designed studies that produce negative results generate information necessary to improve our understanding of causal processes related to disease and treatment, thereby advancing both drug discovery and the efficient application of licensed interventions at the bedside.

Our analysis also points to opportunities for improving the translation process. First, the drug development enterprise needs more effective ways of capturing and utilizing the vast amounts of information it generates. Trials do more than test new interventions; they also deliver evidence on pathophysiological theories and the limits of validated treatments. Most of this information is inaccessible to the broader research community due to non-publication and poor reporting. For example, one of us (JK) and co-workers recently looked at drugs that reached phase 3 trials but were never successfully licensed; results were published for only 37% of trials for these failed drugs (Hakala et al., 2015). Another study showed that only 17% of healthy volunteer phase 1 trials are published (Decullier et al., 2009). Even where trials are published, information probing pathophysiological theories is often withheld. Less than 40% of pharmacodynamics analysis results—which probe pathophysiological theories—within cancer trials are published in full, and what is reported is often difficult to interpret due to incomplete description of methods (Freeman and Kimmelman, 2012). More needs to be done to demand the publication of results from early-phase research, data from trials of products that are not licensed, and data from pharmacodynamics studies conducted within larger research studies.

Companies tend not to publish negative or inconclusive studies or secondary analyses for a number of reasons: such findings can be exploited by competing drug companies; they can complicate a clean narrative for investors; and they can diminish off-label use of a licensed drug. Similar pressures may deter academics from pursuing publication as well. Various private (Green et al., 2015; Peart, 2015) and public initiatives (Collins and Tabak, 2014) have been established to increase the transparency of trials and the release of data: however, given the volume of data that is not captured and the incentives for companies not to disclose certain data, these efforts are not enough. Policy-makers and funding bodies should pursue measures that promote unbiased and prompt publication for unsuccessful translation trajectories. Public funding agencies might also consider withholding the last installments of grants until all the relevant data have been made public, or they might offer small grants for publishing negative results. When hosting privately funded trials, academic medical centers could insist on using contracts that commit sponsors to sharing full datasets with local investigators, and that allow researchers to publish complete datasets when private sponsors do not. Lawmakers might create policies—like extending the period where drug companies can prevent competitors from using their trial data in licensing applications—that provide a financial incentive for publishing trials submitted to drug regulators for new drugs.

Second, researchers and healthcare professionals should better exploit the information generated by unsuccessful translation trajectories. Researchers should pay more attention to negative and inconclusive findings when they are published: researchers tend to cite positive findings more frequently than negative findings, even when there are no obvious differences in the quality of the studies (Greenberg, 2009), and many clinical investigators perceive negative studies as uninformative (Smyth et al., 2011). Yet without comprehensive data about all of the investigations motivated by a common theoretical framework, it is difficult to distinguish unsuccessful translation trajectories that reflect shortcomings in the study from those that are informative about strategies for turning interventions into therapies. Moreover, when studied together, positive and negative studies for a given drug can help clinicians to refine their understanding of the drug and/or the disease it is intended to treat. Journal editors should encourage researchers to include negative and inconclusive findings in their papers and to explain what these findings mean for our understanding of the drug and/or the disease. Ethics committees can also encourage such a shift by demanding that trial brochures discuss any relevant negative and inconclusive findings that have been made public: if nothing else, this will help to distinguish proposed interventions from similar ones that have failed, and may also shed light on why the trial is being conducted on certain subpopulations.

Researchers and healthcare professionals should better exploit the information generated by unsuccessful translation trajectories.

Third, policy makers and researchers should be cautious about policies that aim to accelerate translation by granting licenses for products early in the trial process. Recent Japanese legislation will allow the commercialization of cell-based interventions once their safety has been established in in early-phase studies (Cyranoski, 2013), and a drug for the treatment of lung cancer was recently approved for use in the US after a single phase 1 study (Khozin et al., 2015). As our analysis indicates, such policies harbor liabilities. Large effects in early development are likely to regress in later trials, and truncating the development process means that we will not gather useful information about optimal dosing, diagnostic cut-points, or which subpopulations to use a drug in. Early licensing slows the collection of this evidence by discouraging patient recruitment into trials (Rettig, 2007) and by diminishing incentives for other companies to run trials. The result of such reforms may be an increase in the number of patients exposed to harmful or ineffective interventions, and/or longer timelines for the development of truly effective treatments. Such reforms are also likely to shift the cost of gathering this information from drug developers to patients and healthcare systems.

Until our knowledge of the underlying processes that cause disease improves, the best approach for increasing the efficiency of translation is to maximize the information gained from patient exposure. This means better reporting on carefully planned trials, greater uptake of evidence from unsuccessful translation trajectories, and using failure in drug development to improve the search for or the use of new interventions. Without explicit recognition of the statistical, scientific and ethical factors that limit the rate of drug development, efforts to make translation more efficient risk shifting costs and burdens without producing better options for patients and healthcare systems.
